# THE VA GERIATRIC SCHOLARS PROGRAM IMPACT ON CLINICAL INNOVATIONS: QI COMPONENT IMPACTS OVER THE PAST DECADE

**DOI:** 10.1093/geroni/igae098.0845

**Published:** 2024-12-31

**Authors:** Carol Callaway-Lane

**Affiliations:** Tennessee Valley VA, Nashville, Tennessee, United States

## Abstract

Since 2009 one of the key components of the Geriatric Scholars Program (GSP) has been the Quality Improvement Workshop and Practicum, offering the fundamentals of improvement science to scholars and guidance through real-time improvement projects within rural clinical settings. The GSP has offered this component to a wide range of providers, starting in 2009 with prescribing providers and pharmacists, expanding to an interprofessional consortium including the initial professions as well as social work, psychiatry, psychology, and functional therapists and assistants. Impact on the aging Veteran population is demonstrated through completion of improvement projects at the local level with 79 national presentations completed from 2010 - 2024. This session will explore the evolution of the QI Program over the past decade and how the program has adapted to changes in audience type based on clinical training, presentation format for the workshop material, and impact on project topic and completion rates over time. Improvement projects throughout the years and their impact on the national push for High Reliability Organization (HRO) status will also be discussed.

